# Tracking post-infectious fatigue in clinic using routine Lab tests

**DOI:** 10.1186/s12887-016-0596-8

**Published:** 2016-04-26

**Authors:** Jeanna M. Harvey, Gordon Broderick, Alanna Bowie, Zachary M. Barnes, Ben Z. Katz, Maurice R. G. O’Gorman, Suzanne D. Vernon, Mary Ann Fletcher, Nancy G. Klimas, Renee Taylor

**Affiliations:** Department of Medicine, University of Miami, Miami, FL USA; Institute for Neuro Immune Medicine, Nova Southeastern University, University Park Plaza, 3440 South University, Fort Lauderdale, 33328 FL USA; University of Alberta, Edmonton, AB Canada; Ann & Robert H Lurie Children’s Hospital of Chicago, Chicago, IL USA; Children’s Hospital Los Angeles, Los Angeles, CA USA; Solve ME/CFS Initiative, Charlotte, NC USA; University of Illinois at Chicago, Chicago, IL USA

**Keywords:** Blood glucose, ACTH, Estradiol, Neutrophil count, Free thyroxin, Salivary cortisol, Classification models, Infectious mononucleosis, EBV, Chronic fatigue

## Abstract

**Background:**

While biomarkers for chronic fatigue syndrome (CFS) are beginning to emerge they typically require a highly specialized clinical laboratory. We hypothesized that subsets of commonly measured laboratory markers used in combination could support the diagnosis of post-infectious CFS (PI-CFS) in adolescents following infectious mononucleosis (IM) and help determine who might develop persistence of symptoms.

**Methods:**

Routine clinical laboratory markers were collected prospectively in 301 mono-spot positive adolescents, 4 % of whom developed CFS (*n* = 13). At 6, 12, and 24 months post-diagnosis with IM, 59 standard tests were performed including metabolic profiling, liver enzyme panel, hormone profiles, complete blood count (CBC), differential white blood count (WBC), salivary cortisol, and urinalysis. Classification models separating PI-CFS from controls were constructed at each time point using stepwise subset selection.

**Results:**

Lower ACTH levels at 6 months post-IM diagnosis were highly predictive of CFS (AUC *p* = 0.02). ACTH levels in CFS overlapped with healthy controls at 12 months, but again showed a trend towards a deficiency at 24 months. Conversely, estradiol levels depart significantly from normal at 12 months only to recover at 24 months (AUC *p* = 0.02). Finally, relative neutrophil count showed a significant departure from normal at 24 months in CFS (AUC *p* = 0.01). Expression of these markers evolved differently over time between groups.

**Conclusions:**

Preliminary results suggest that serial assessment of stress and sex hormones as well as the relative proportion of innate immune cells measured using standard clinical laboratory tests may support the diagnosis of PI-CFS in adolescents with IM.

**Electronic supplementary material:**

The online version of this article (doi:10.1186/s12887-016-0596-8) contains supplementary material, which is available to authorized users.

## Background

Chronic Fatigue Syndrome (CFS) is a complex, multi-symptom illness involving persistent fatigue, musculoskeletal symptoms, as well as cognitive impairment [1]. Studies show that CFS affects between 1 and 4 million individuals [2, 3] and costs an estimated $35 billion per year in health care and lost productivity [4, 5]. CFS affects roughly 0.2 % of the general adolescent population [6] and is a major cause of educational disruption [7–10]. While viral serology is not necessary for diagnosis, CFS follows infection in at least a subset of cases [1, 11]. In adults, symptom onset coincided with apparent infection in up to 72 % of cases; [12] in adolescents, infectious mononucleosis (IM) is the most commonly reported antecedent infection [9, 13–15]. The reported incidence of CFS following IM is 9-12 % [16–19]. Females appear more susceptible than males [10, 16, 20, 21]. Consistent with this, our group reported previously that 13, 7 and 4 % of adolescents met criteria for CFS at 6, 12 and 24 months respectively post-IM, and at 6 months post-IM, 90 % of the subjects with CFS were female, while at 12 and 24 months, 100 % were female [22].

Diagnosis of CFS currently relies on exclusion of other medical or psychological causes [1, 23, 24]. Currently there are no validated diagnostic test, although Klimas et al. [25] have reviewed some candidate biomarkers. Confirming many but not all past reports, Brenu et al. [26] and Fletcher et al. [27] found natural killer (NK) cell cytotoxicity to be low. Fletcher et al. [28] also found evidence that Neuropeptide Y, an immunologically active neurotransmitter, was elevated in subjects with CFS and correlated with symptom severity. Mild hypocortisolism with attenuated diurnal variation has also been reported in some patients with CFS [29]. In addition to the early identification of potential individual candidate markers, studies using multiplex methods have reported broader surveys and more detailed analyses. For example, while Curriu et al. [30] found similar cell counts for T, B, and NK cell populations in general in healthy individuals and subjects with CFS (*n* = 22), the latter showed significant increases in the T-helper and regulatory T cell subsets. NK cells also showed higher levels of specific activation markers (e.g., CD69). Suggesting altered patterns of immune signaling, Broderick et al. [31] found that a subset of 5 cytokines used in combination supported an accuracy of better than 80 % at a confidence level of 0.95 in separating subjects with post-IM CFS versus recovered controls.

In the present work we focused on common clinical laboratory assays measured longitudinally to explore whether a combination of such markers could act as a signature for CFS following IM. We reviewed commonly recorded laboratory data collected during a 2-year prospective study of 301 adolescents with mono-spot positive IM conducted by Katz et al. [22] in the greater Chicago area. In this study 4 % of subjects met criteria for CFS per the Jason et al. revision [32] of the Fukuda criteria [1]. Fifty-nine standard blood, saliva, and urine tests, such as CBC with differential, metabolic profiling, and urinalysis, were performed at each time point. Linear classification models separating post-IM CFS from controls were constructed at each time point based on subsets of these markers selected iteratively. While the vast majority of these markers were uninformative, changes in ACTH, estradiol, and relative neutrophil count across the 2-year period were discriminatory for post-infectious CFS (PI-CFS) with an accuracy ranging from 72 to 84 %.

### Methods

#### Cohort and clinical assessment

A total of 301 adolescents, ages 12–18, with a diagnosis of monospot positive acute IM were recruited on a referral basis from the greater Chicago area via school nurses, pediatric practices, and the Virology Laboratory of Children’s Memorial Hospital (now the Ann & Robert H Lurie Children’s Hospital of Chicago) between September of 2004 and November of 2007. The presumption was that most cases of monospot-positive acute IM were caused by Epstein-Barr virus (EBV) infection. Of the 301 initially enrolled, 286 adolescents who were not fully recovered as well as those who recovered uneventfully (controls) successfully completed the telephone-based interview using the CFS Screening Questionnaire [33] and underwent evaluation at 6, 12, and 24 months post-IM. Of these subjects, 13, 7, and 4 % (*n* = 13) respectively met the Jason et al. revision [32] of the Fukuda [1] case definition for CFS. The former also incorporates elements of the Canadian case definition reported in Carruthers et al. [23]. Ninety percent of subjects who met the CFS case definition at 6 months were female, and at 12 and 24 months, 100 % of subjects who met the case definition were female. At 6, 12, and 24 months post-IM, blood, saliva, and urine samples were collected from cases and controls for laboratory analysis. Details of the full cohort are reported in Katz et al. [22]. In the present study we focus on the specific subset of 13 female adolescents, representing 4 % of the initial cohort of over 300 adolescents, who fit the case definition of CFS at 6, 12 and 24 months following diagnosis with IM. These adolescents were compared to *n* = 12 female control subjects, case-matched on the basis of gender, age (+/− 1 year, with 1 exception) and Tanner breast and pubic hair stage (4 or 5, with one exception), who recovered normally following the same infection. All subjects were menarchal. None of the patients were on significant, chronic medications. Five were on no medications. Four reported occasional inhalers for asthma, or Advil. Three were on birth control. One patient was on methylphenidate (Concerta), one had a history of clonidine use in the past and one reported 1 or 2 doses of Xanax per year because of fear of flying.

#### Ethics statements

All aspects of the study were approved by the Institutional Review Boards of Children’s Memorial Research Center (now the Stanley Manne Research Institute) and the College of Applied Sciences of the University of Illinois at Chicago. Written informed consent was obtained from all participants age 18 years or older. For those subjects less than 18 years of age, written informed consent was obtained from their guardians, coupled with mandatory child assent. Secondary analysis of the biological samples and data was reviewed and approved by the Institutional Review Board of the University of Alberta.

#### Laboratory measurements

Morning fasting blood samples were collected into ethylene diamine tetra acetic acid (EDTA) anticoagulant tubes from cases and controls at 6, 12, and 24 months following the diagnosis of monospot positive, acute IM. Where appropriate plasma was separated within 2 h of collection and stored at -80oC until assayed. A total of 59 standard laboratory tests were performed according to Good Clinical Laboratory Practice standards on blood and urine, including complete blood count with differential, metabolic profiling, erythrocyte sedimentation rate, liver enzymes, thyroid, adrenal, and sex hormone profiling, and urinalysis. Cortisol was isolated from passive drool with a solid phase extraction protocol using a Micromass Quattro Micro triple-quadrupole mass spectrometer equipped with a Z-spray ion source and a Waters 2795 Alliance HT HPLC system as described in Katz et al. [34] Summary statistics for the expression of these markers are shown in Additional file 1: Tables S1a-d.

#### Statistical analyses

To assess the significance of changes in the mean expression of individual markers occurring across each of these patient groups versus recovering controls, we used a standard parametric *t*-test after performing a logarithmic transformation. In conjunction with this, a nonparametric Wilcoxon rank-sum test was used to compare the difference in group-wise median expression for each marker at each time point. The significance of the effects for time and time x group interactions were assessed using a conventional 2-way ANOVA and verified further using a regression-based repeated measures analysis. These calculations were performed using the *annovan* as well as the *fitrm* and *ranovatbl* functions available in the MatLab Statistics Toolbox (The MathWorks, Inc., Natick, MA).

Individual markers were assessed using different laboratory assays. In conventional multiple testing problems this heteroscedasticity or non-uniform variance can be removed by rescaling. However, when the null hypothesis involves correlated markers as it does here, such a rescaling step is known to distort the result and methods to address this issue still constitute an active field of research [35]. Thus, raw null probability *p*-values are more appropriate in this situation determining the significance of group-wise changes. Nevertheless, we estimated false discovery rates (FDR) using both the more exploratory measure proposed by Storey [36] and the more conservative measure of Benjamini and Hochberg [37]. In the case of the Storey [36] FDR, we estimated the tuning parameter lambda (λ) using both a bootstrap method and a polynomial regression. We found the latter to support more conservative estimates of FDR in this data set. FDR calculations were performed using the *mafdr* function available in the MatLab Statistics Toolbox (The MathWorks, Inc., Natick, MA).

In addition, we examined patterns of expression in multiple markers at the level of individual subjects to address the non-homogeneous nature of these groups. This was done by constructing linear discriminant classification models using a stepwise feature selection method to mitigate the effect of cross-correlation between markers [38]. Such regression models favor marker subsets that are minimally redundant [39]. Model terms were selected sequentially based on their respective partial-F test values. Markers with a null probability p(partial F) < 0.05 were selected for recruitment into the regression model while those currently in the model but showing a revised p(partial F) > 0.10 were removed. In the resulting model, an observed row × from the sample set is classified into group I rather than group J if 0 < B_0_ + x*B, where the coefficient vector B and intercept vector B_0_ are estimated from the data. All markers were normalized a priori using a conventional z-score to a mean value of 0 and a variance of 1.0 with observations at 6 months serving as the reference datum. Classifiers were evaluated based on their overall error rate (incorrectly classified/total classified) in assigning subjects to their proper diagnostic group. Detection sensitivity and assignment specificity were then computed over a range of threshold values B_0_ to produce a receiver-operator characteristic (ROC) curve [40]. These calculations were performed using the *classify, classperf, rankfeatures and randfeatures* functions available in the MatLab Statistics Toolbox and the MatLab Bioinformatics Toolbox (The MathWorks, Inc., Natick, MA) as well as with the SPSS Statistics Release 21.0 software package (IBM, New York, NY). For a further introduction and review of these statistical measures as they might apply more to clinical practice we refer the reader to [41, 42].

## Results

The results of the parametric t-tests and nonparametric Wilcoxon rank-sum tests performed on all standard blood work, salivary tests, and urinalysis measures are presented in Additional file 1: Tables S1a-d. At 6 months, those with PI-CFS were found to have depressed glucose (*p* = 0.03, ranksum *p* = 0.05) and depressed ACTH (*p* = 0.01, ranksum *p* = 0.02) compared with controls. At 12 months, those with PI-CFS were found to have depressed estradiol compared with controls (*p* = 0.01, ranksum *p* = 0.02), and at 24 months, those with PI-CFS had higher levels of neutrophils compared with controls (*p* =0.02, ranksum *p* = 0.01). Although this did not meet statistical significance, we also found elevated free thyroxine (T4) at both 6 (*p* = 0.07) and 12 months (*p* = 0.08). In this group-wise comparison conducted separately at each time point none of the *p*-values obtained, whether by *t* test or ranksum test, corresponded to a FDR of less than 0.20. This suggests that if values are considered independent from one visit to the next the probability of an incidental finding is high. However when analyzing the full time course using a 2-way ANOVA (Additional file 2: Table S2), we found a significant group effect for elevated free thyroxine (T4) (*p* = 0.01; FDR = 0.01, FDR _BH_ = 0.26) and depressed morning salivary cortisol (*p* = 0.03; FDR = 0.02, FDR _BH_ = 0.29) as well as significant time (*p* = 0.01; FDR = 0.02, FDR _BH_ = 0.11) and group effects for ACTH (*p* = 0.01; FDR = 0.03, FDR _BH_ = 0.26). All three of these markers, namely free thyroxine (T4), morning salivary cortisol and ACTH demonstrated group effects that satisfied a FDR < 0.05 based on Storey [36] using a polynomial estimate for the tuning factor lambda (λ). In none of the markers studied were we able to find a change in the slope of the trend from one subject group to the next (i.e., time × group effect). These results were largely confirmed when performing a complementary analysis using a regression-based repeated measure model (Additional file 3: Tables S3a - d) with the exception of a few minor differences in the significance of the time effect only. Once again no significant time x group effects were identified.

When linear regression models were applied, one variable, unique at each time point, was selected as being the most suitable to distinguish between individuals recovering normally and those who progressed to PI-CFS. At 6, 12, and 24 months, ACTH, estradiol, and neutrophils, respectively, were highly discriminatory for PI-CFS. As shown in Table 1, at 6 months, the model based on depressed ACTH levels produced an accuracy of 72 %, a negative predictive value (NPV) of 73 %, positive predictive value (PPV) of 71 %, specificity of 67 %, and sensitivity of 77 %. In comparison, low glucose levels supported a slightly higher PPV (75 %) and specificity (75 %), but lower NPV (69 %) and sensitivity (69 %). Similarly at 12 months, the model based on depressed estradiol alone produced an accuracy of 76 %, a NPV of 75 %, a PPV of 77 %, with a specificity of 75 %, and a sensitivity of 77 %. The classification accuracy increases as the sample time approaches 24 months. When applied to subject classification at 24 months, the model based on increased neutrophil count produced an accuracy of 84 % corresponding to a NPV of 83 %, PPV of 85 %, specificity of 83 %, and sensitivity of 85 %.

**Table 1:** Identification and performance of linear classification models. Classification of PI-CFS and healthy recovered control (RC) subjects at 6, 12 and 24 months post-diagnosis with IM using linear discriminant models with terms identified as optimal at each time point based on classical stepwise variable selection with the exception of classification based on glucose levels at 6 months which was specified based on differential expression

Model	Assigned RC	Assigned PI-FS	Total		Accuracy	NPV	PPV	Specificity	Sensitivity
6 months									
ACTH	8	4	12	True RC	0.72	0.73	0.71	0.67	0.77
	3	10	13	True PI-CFS					
Glucose	9	3	12	True RC	0.72	0.69	0.75	0.75	0.69
	4	9	13	True PI-CFS					
12 months									
Estradiol	9	3	12	True RC	0.76	0.75	0.77	0.75	0.77
	3	10	13	True PI-CFS					
24 months									
Neutrophils	10	2	12	True RC	0.84	0.83	0.85	0.83	0.85
	2	11	13	True PI-CFS					

**Fig. 1:**
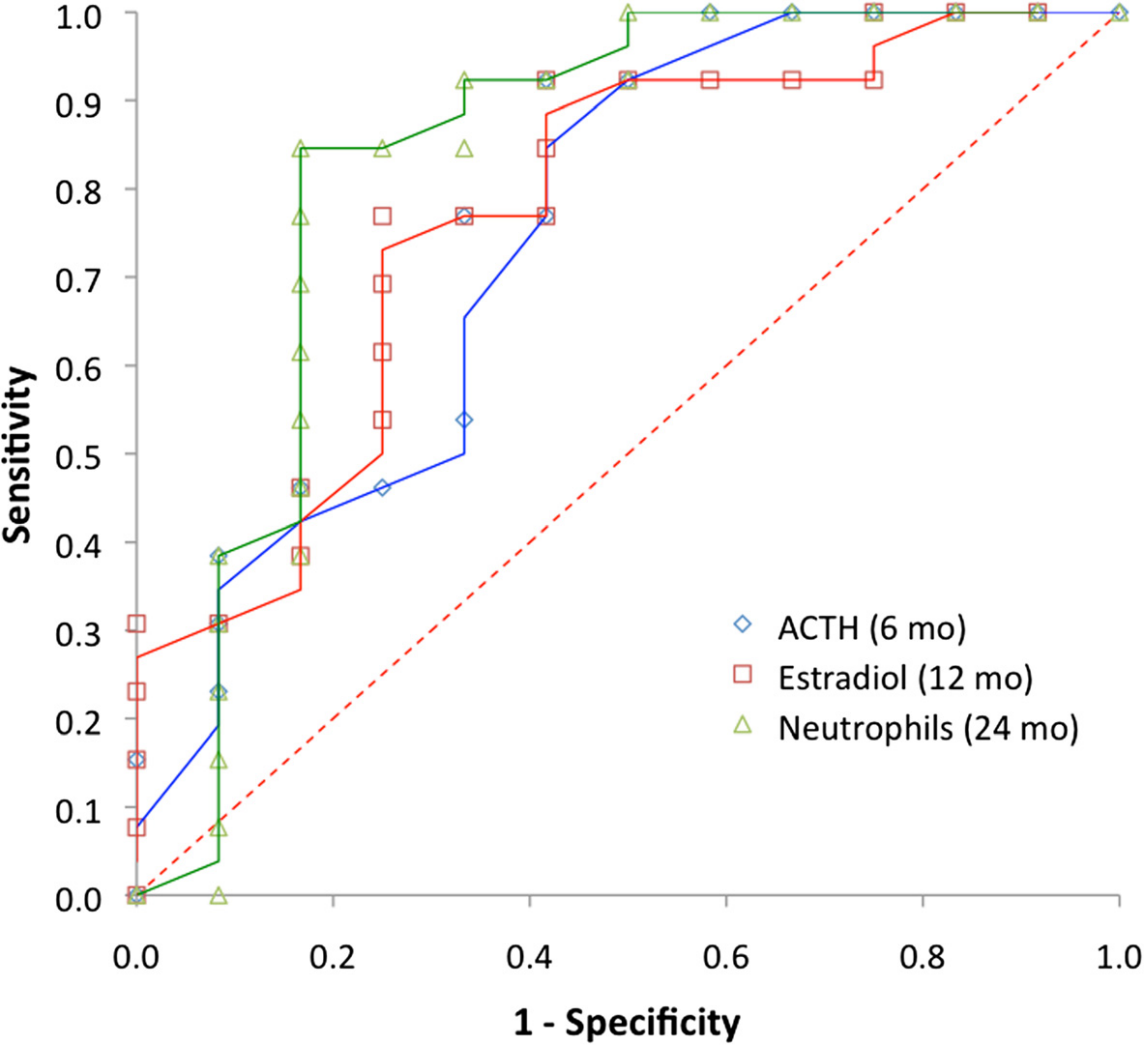
Performance of subject classification based on a minimal set of standard clinical assays. ROC curves for classification of subjects who went on to develop post-infectious chronic fatigue syndrome and subjects who recovered normally using a linear discriminant model selected as optimal based on data collected at 6, 12 and 24-month time points using a stepwise selection method. Coefficients were tuned based on data collected at each time point only

**Fig. 2:**
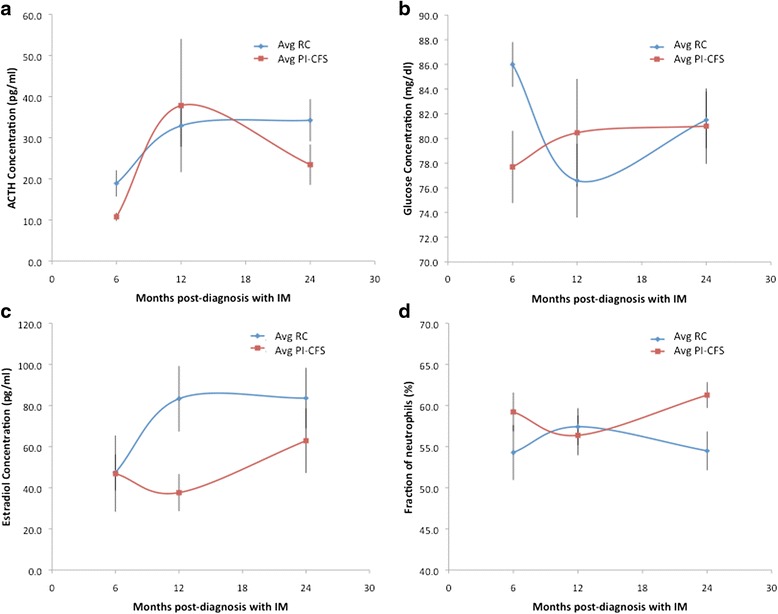
Time course progression in expression of key discriminatory makers. Evolution of ACTH concentration (**a**), glucose concentration (**b**), estradiol concentration (**c**) and neutrophil count (**d**) in blood collected at 6, 12 and 24 months post-diagnosis with IM in subjects who went on to develop post-infectious chronic fatigue syndrome (PI-CFS; red square) and normally recovered control subjects (RC; blue triangle)

Table 2 shows a summary of receiver operating characteristic (ROC) performance for classification of PI-CFS subjects at 6, 12, and 24 months post-diagnosis of IM using linear discriminant models with terms selected as optimal at each time point. Classification for the eventual onset of post-IM CFS at the 2-year time point based on ACTH levels measured at 6 months produced an area under the curve (AUC) of 0.77 (*p* = 0.02). In comparison, glucose measured at 6 months only supports an AUC value of 0.73 (*p* = 0.05). Estradiol levels measured at 12 months produced a classification for illness at 24 months with an AUC value of 0.78 (*p* = 0.02). Finally, illness at 24 months was predicted based on the relative level of neutrophils at that same time point with a highly significant value for the AUC of 0.83 (*p* = 0.01). The ROC curves produced based on measures taken at each time point are presented in Fig. 1 for the markers selected by stepwise regression, namely ACTH, estradiol and relative neutrophil count. The evolution across the 2-year period of all 4 differentially expressed markers is presented in Fig. 2.

**Table 2:** Summary of receiver operating characteristic (ROC) performance. Area under the classification curve (AUC) for assignment of PI-CFS subjects at 6, 12 and 24 months post-diagnosis with IM using a linear discriminant models with terms selected as optimal at each time point using s stepwise selection method. Classification performance based on glucose levels measured at 6 months is included for comparison and was specified on the basis of t and ranksum tests

	Area	Std. error^a^	Asymptotic Sig.^b^	Asymptotic 95 % Conf. interval	
Model				Lower bound	Upper bound
ACTH (6 months)	0.77	0.10	0.02	0.58	0.96
Glucose (6 months)^c^	0.73	0.11	0.05	0.52	0.95
Estradiol (12 months)	0.78	0.09	0.02	0.60	0.97
Neutrophil (24 months)	0.83	0.09	0.01	0.64	1.00

The test result variable(s): acth1 has at least one tie between the positive actual state group and the negative actual state group. Statistics may be biased

^a^Under the nonparametric assumption

^b^Null hypothesis: true area = 0.5

^c^Model specified on the basis of t and ranksum tests, not as a result of stepwise selection

## Discussion

The diagnosis of CFS currently relies on the exclusion of other medical and psychiatric diseases. We sought to determine if standard laboratory analysis of blood, salivary, and urine markers used alone or in combination might support the identification of individuals likely to develop PI-CFS. In the adolescent population studied we found significantly lower levels of glucose and ACTH 6 months following IM in individuals who 18 months later would continue to suffer with persistent symptoms. Glucose was not selected as a distinguishing variable using stepwise regression even though it displayed similar predictive power because of a strong overlap with similar changes in ACTH. Changes in glucose homeostasis during infection are context-dependent and poorly understood [43] with cytokines such as IL-6 reportedly promoting hypoglycemia during acute LPS-induced inflammation [44]. Interestingly relative expression of the latter emerged as a distinguishing feature for this illness group in our previous work [31]. In addition, significantly lower levels of estradiol and increased relative neutrophil count were also observed 12 and 24 months post-IM (Table 1). The accuracy of each individual marker in identifying individuals who develop PI-CFS at 24 months ranged between 72-84 %. The fact that different markers discriminated better at different times might highlight the dynamic nature of the immune and endocrine responses to this insult, which could be characterized as progressive. In several markers a persistent offset appears to be acquired quite early however the nonlinear nature of these changes is not easily captured by traditional linear analysis, e.g., the 2-way ANOVA employed herein. Because of this, single assessments will be of limited value while serial assessments within the first year may be much better suited for aiding in diagnosis of these subjects (e.g., serial estradiol measurements).

CFS is a complex constellation of symptoms [25]. Cannon et al. [45] found sex hormone driven changes in neutrophil count characteristic of CFS. The latter reported that while neutrophil count normally increases from the follicular to luteal phase in healthy women, it persists at luteal levels in CFS. Niblett et al. [46] also found that the neutrophil count and ratio of neutrophils to lymphocytes were both increased in CFS subjects. Discrepancies in neutrophil function have also been observed in some subjects with CFS with an overabundance of pre-apoptotic cells [47, 48]. Significant alterations in NK cell signaling and function have also been observed in some populations with CFS such as in our previous work in older women, where the elimination of target erythroleukemic K562 cells by NK cells was significantly impaired in CFS [27]. However Katz et al. [34] studying a similar population to that reported here found normal NK cell number and function in adolescents with CFS following IM. In addition to differences in the ages of the subjects between the two previously cited studies, there were also differences in methodology; the latter assessed cytotoxic function of NK cell isolates while the former studied NK cell function in a mixed lymphocyte population.

Involvement of sex-hormones in CFS is also consistent with our earlier work [49] and that of others, in which women were significantly more affected by this illness than men [10, 16, 22]. Our recent analysis using a theoretical model of regulatory physiology linking the hypothalamic-pituitary-adrenal (HPA) axis, the hypothalamic-pituitary-gonadal (HPG) and the peripheral immune system have shown that these interacting systems are especially prone in women to support chronically depressed cortisol levels and a persistent inflammatory signature consistent in some CFS populations [50].

Dysregulation of normal hypothalamic-pituitary-adrenal (HPA) axis function has been reported previously in patients with CFS [51]. Again, in our previous study from this population, we found no evidence of consistently depressed salivary cortisol in adolescents with CFS compared with recovered controls when individual time points were compared, although 3 of 9 subjects with CFS did have depressed morning cortisol (versus 1 of 9 controls). We concluded that depressed salivary cortisol may be an uncommon factor in CFS as even in those cases where it might play a role it did not persist despite persistence of symptoms [34]. In the current report, we found evidence of a group effect for depressed morning salivary cortisol as well as possibly other elements of hypothalamic-pituitary-thyroid (HPT) dysregulation with elevated free T4 at both 6 (*p* = 0.07) and 12 months (*p* = 0.08), which trended towards significance. These findings are also consistent with Fuite et al. [52] who found evidence of re-modeling in neuro-endocrine and immune signaling in CFS. Specifically, results suggested a decreased cohesiveness in HPA axis response that coincided with a characteristic co-expression of immune markers with thyroid hormones in a larger group (*n* = 39) female CFS subjects. Changes in HPA axis feedback dynamics have also been reported recently by Aschbacher et al. [51] who describe an abnormal increase in negative feedback along the HPA axis in CFS. If examined individually, the observed decreases in these markers, while statistically significant, may escape biological significance. For example, estradiol levels from 10 to 260 are all normal for either the luteal or follicular phases of the menstrual cycle. Our study is an ad hoc analysis of prospectively collected data. If confirmed prospectively, depressed ACTH and possibly glucose might offer an objective way to confirm the diagnosis of CFS at 6 months following IM, while persistence of increased T4 and decreased morning salivary cortisol coupled with decreased estradiol at 12 months and increased neutrophil counts at 24 months might provide objective evidence for the persistence of CFS. Whether any of these findings would be helpful earlier than 6 months following IM is not known.

Limitations of this exploratory study include the small number of subjects (*n* = 13) and the relatively coarse sampling interval; nonetheless these were prospectively collected data from a study of PI-CFS with a well-defined trigger (IM). While these findings may not be applicable to the entire CFS population, a significant subset thereof reports onset coinciding with infectious illness. Though it is necessary validate these findings in a larger cohort, these initial results support the potential opportunity to identify individuals most at risk for developing CFS following IM and who might benefit from a more pro-active treatment. We purposely limited our assessment to common clinical markers in blood, saliva, and urine; while these readily available tests may not have the resolution to make a diagnosis, they may be sufficiently robust to support one. Indeed, additional means of confirmation using more focused laboratory assays as well as physiologic challenges to amplify these differences are being studied [53]. For example, in applying a maximal exercise challenge to this cohort our group found significantly higher oxygen consumption, work slope, and peak oxygen pulse in recovered subjects versus patients diagnosed with PI-CFS at 6 months after IM [54]. Other groups are reporting that such differences increase several fold in a follow-up challenge performed 24 h later [55] suggesting that a stress test may be much more revealing than a standard assessment. These limitations not withstanding, we found evidence that the onset of PI-CFS may be associated with the expression of specific patterns of markers in standard blood work. Whether these markers are etiologically related to CFS also remains to be determined and further studies should include at least one comparator illness common to this age group.

## Conclusions

In a first exploratory analysis of a clinical data set we found that assessment of stress and sex hormones as well as the relative proportion of innate immune cells using standard clinical laboratory tests may help support the diagnosis of PI-CFS in adolescents with IM. Specifically, early course deviations in ACTH and possibly glucose along with subsequent differences in the levels of morning salivary cortisol, T4 and estradiol might provide objective evidence for the onset and persistence of CFS. As the relative deviations in these markers evolve over time, their contribution to accuracy and robustness of classification is greatly improved if they are recorded longitudinally.

## Additional files

Additional file 1: Table S1a. Summary descriptive statistics of clinical assays. Mean and standard deviation () of the conventional complete metabolic profiling in blood at 6, 12 and 24 months following diagnosis with IM. **Table S1b**. Summary descriptive statistics of clinical assays. Mean and standard deviation () of the conventional complete blood count (CBC) profiling in blood at 6, 12 and 24 months following diagnosis with IM. **Table S1c**. Summary descriptive statistics of clinical assays. Mean and standard deviation () of the conventional differential blood count profiling in blood at 6, 12 and 24 months following diagnosis with IM. Basophil count was not considered due to a large proportion of missing values. **Table S1d**. Summary descriptive statistics of clinical assays. Mean and standard deviation () of the conventional endocrine profiling in blood with urine specific gravity and pH at 6, 12 and 24 months following diagnosis with IM. (DOC 149 kb) Additional file 2: Table S2a. Summary of 2-way ANOVA. Significance of time, group and time x group effects of conventional metabolic profiling in blood across 6, 12 and 24 months following diagnosis with IM. False discovery rates (FDR) were based on Storey [36] using the bootstrap (boot) and the polynomial (poly) fit methods to estimate lambda, as well as on by Benjamini and Hochberg [37] (BH). **Table S2b**. Summary of 2-way ANOVA. Significance of time, group and time x group effects for complete blood count (CBC) profiling in blood across 6, 12 and 24 months following diagnosis with IM. False discovery rates (FDR) were based on Storey [36] using the bootstrap (boot) and the polynomial (poly) fit methods to estimate lambda, as well as on by Benjamini and Hochberg [37] (BH). **Table S2c**. Summary of 2-way ANOVA. Significance of time, group and time x group effects for conventional differential blood count profiling in blood across 6, 12 and 24 months following diagnosis with IM. False discovery rates (FDR) were based on Storey [36] using the bootstrap (boot) and the polynomial (poly) fit methods to estimate lambda, as well as on by Benjamini and Hochberg [37] (BH). Basophil count was not considered due to a large proportion of missing values. **Table S2d**. Summary of 2-way ANOVA. Significance of time, group and time x group effects for standard endocrine profiling in blood with urine specific gravity and pH across 6, 12 and 24 months following diagnosis with IM False discovery rates (FDR) were based on Storey [36] using the bootstrap (boot) and the polynomial (poly) fit methods to estimate lambda, as well as on by Benjamini and Hochberg [37] (BH). (DOC 150 kb) Additional file 3: Table S3a,b,c and d. Supplemental tables containing summary statistics describing the significance and probability of false discovery for the effects of time and the time x group interaction for each of the clinical markers using a regression-based repeated measures model. (XLSX 48 kb)

## Abbreviations

ACTH: adrenocorticotropic hormone
EBV: Epstein-Barr virus
IM: infectious mononucleosis
ME/CFS: myalgic encephalomyelitis/chronic fatigue syndrome
