# Supplementary material for: Tracking post-infectious fatigue in clinic using routine Lab tests
Source: BMC Pediatr. 2016 Apr 26;16:54. doi: 10.1186/s12887-016-0596-8 (PMC4847210; doi:10.1186/s12887-016-0596-8)
Supplement: Additional file 1: Table S1a. — Summary descriptive statistics of clinical assays. Mean and standard deviation () of the conventional complete metabolic profiling in blood at 6, 12 and 24 months following diagnosis with IM. Table S1b. Summary descriptive statistics of clinical assays. Mean and standard deviation () of the conventional complete blood count (CBC) profiling in blood at 6, 12 and 24 months following diagnosis with IM. Table S1c. Summary descriptive statistics of clinical assays. Mean and standard deviation () of the conventional differential blood count profiling in blood at 6, 12 and 24 months following diagnosis with IM. Basophil count was not considered due to a large proportion of missing values. Table S1d. Summary descriptive statistics of clinical assays. Mean and standard deviation () of the conventional endocrine profiling in blood with urine specific gravity and pH at 6, 12 and 24 months following diagnosis with IM. (DOC 149 kb) [file 12887_2016_596_MOESM1_ESM.doc]

**Table S1a.** **Summary descriptive statistics of clinical assays**. Mean and standard deviation ( ) of the conventional complete metabolic profiling in blood at 6, 12 and 24 months following diagnosis with IM.

|  | **6 mo** |  |  |  |  | **12 mo** |  |  |  |  | **24 mo** |  |  |  |
| --- | --- | --- | --- | --- | --- | --- | --- | --- | --- | --- | --- | --- | --- | --- |
| Marker | RC | PI-CFS | p t test | p ranksum |  | RC | PI-CFS | p t test | p ranksum |  | RC | PI-CFS | p t test | p ranksum |
| Sodium | 137.92 (1.68) | 138.38 (1.33) | 0.44 | 0.44 |  | 137.83 (1.53) | 138.38 (1.19) | 0.32 | 0.37 |  | 138.08 (2.07) | 137.77 (1.30) | 0.65 | 0.82 |
| Potassium | 3.98 (0.44) | 3.90 (0.29) | 0.58 | 0.68 |  | 4.08 (0.24) | 4.09 (0.24) | 0.86 | 0.93 |  | 4.67 (1.65) | 4.37 (0.26) | 0.53 | 0.21 |
| Chloride | 104.83 (1.80) | 105.00 (2.58) | 0.85 | 0.74 |  | 104.58 (1.68) | 105.31 (1.84) | 0.32 | 0.21 |  | 105.50 (2.65) | 106.38 (2.50) | 0.40 | 0.36 |
| Carbon Dioxide | 25.19 (1.91) | 24.77 (1.81) | 0.58 | 0.55 |  | 24.06 (2.10) | 24.19 (1.75) | 0.86 | 0.91 |  | 24.23 (4.67) | 23.60 (1.97) | 0.66 | 0.81 |
| Glucose | 86.00 (6.30) | 77.69 (10.55) | *0.03* | *0.05* |  | 76.58 (10.37) | 80.46 (15.75) | 0.48 | 0.37 |  | 81.50 (7.96) | 81.00 (11.01) | 0.90 | 0.72 |
| BUN | 9.33 (1.61) | 8.54 (2.76) | 0.39 | 0.47 |  | 11.17 (3.13) | 9.62 (2.36) | 0.17 | 0.38 |  | 10.67 (2.57) | 9.69 (1.75) | 0.28 | 0.35 |
| Creatinine | 0.73 (0.11) | 0.69 (0.10) | 0.34 | 0.39 |  | 0.77 (0.11) | 0.76 (0.13) | 0.92 | 0.93 |  | 0.75 (0.08) | 0.73 (0.12) | 0.64 | 0.75 |
| Calcium | 9.43 (0.35) | 9.42 (0.25) | 0.98 | 0.98 |  | 9.50 (0.27) | 9.35 (0.27) | 0.17 | 0.21 |  | 9.45 (0.30) | 9.31 (0.27) | 0.23 | 0.32 |
| Total Protein, Serum | 6.74 (0.42) | 6.86 (0.31) | 0.42 | 0.72 |  | 6.94 (0.38) | 6.67 (0.51) | 0.15 | 0.24 |  | 6.78 (0.31) | 6.76 (0.31) | 0.86 | 0.91 |
| Albumin | 3.94 (0.29) | 4.02 (0.24) | 0.45 | 0.56 |  | 4.09 (0.32) | 3.95 (0.34) | 0.30 | 0.18 |  | 3.98 (0.38) | 3.92 (0.32) | 0.65 | 0.64 |
| Bilirubin, Total | 0.90 (0.39) | 0.78 (0.37) | 0.46 | 0.30 |  | 0.86 (0.32) | 0.73 (0.35) | 0.35 | 0.23 |  | 0.78 (0.27) | 0.66 (0.12) | 0.19 | 0.32 |
| Alkaline Phosphatase | 62.50 (11.49) | 66.92 (14.37) | 0.41 | 0.41 |  | 69.08 (17.16) | 68.23 (14.04) | 0.89 | 0.94 |  | 61.00 (11.26) | 60.92 (12.17) | 0.99 | 1.00 |
| ALT (SGPT) | 15.83 (4.17) | 16.00 (4.97) | 0.93 | 0.83 |  | 15.83 (2.95) | 17.15 (8.06) | 0.60 | 0.96 |  | 15.00 (3.59) | 15.77 (3.24) | 0.58 | 0.49 |
| AST (SGOT) | 22.08 (3.70) | 20.38 (4.27) | 0.30 | 0.28 |  | 21.92 (3.55) | 20.54 (5.64) | 0.48 | 0.20 |  | 23.17 (8.68) | 21.46 (3.57) | 0.52 | 0.93 |

**Table S1b**. **Summary descriptive statistics of clinical assays.** Mean and standard deviation () of the conventional complete blood count (CBC) profiling in blood at 6, 12 and 24 months following diagnosis with IM

|  | **6 mo** |  |  |  |  | **12 mo** |  |  |  |  | **24 mo** |  |  |  |
| --- | --- | --- | --- | --- | --- | --- | --- | --- | --- | --- | --- | --- | --- | --- |
| Marker | RC | PI-CFS | p t test | p ranksum |  | RC | PI-CFS | p t test | p ranksum |  | RC | PI-CFS | p t test | p ranksum |
| CBC-White Blood cells | 6.00 (1.49) | 6.78 (2.96) | 0.42 | 0.98 |  | 7.59 (2.59) | 6.57 (2.35) | 0.31 | 0.29 |  | 9.44 (9.34) | 7.01 (2.08) | 0.75 | 0.71 |
| CBC-Red Blood Cells | 4.73 (0.23) | 4.62 (0.31) | 0.32 | 0.51 |  | 4.84 (0.45) | 4.72 (0.34) | 0.45 | 0.59 |  | 4.71 (0.29) | 4.61 (0.24) | 0.32 | 0.30 |
| CBC-Hemoglobin | 13.63 (1.05) | 13.62 (1.07) | 1.00 | 0.87 |  | 14.07 (0.74) | 13.85 (0.50) | 0.41 | 0.45 |  | 13.73 (0.81) | 13.83 (0.74) | 0.74 | 0.85 |
| CBC-Hematocrit | 39.45 (2.54) | 40.11 (3.23) | 0.58 | 0.50 |  | 40.37 (2.17) | 40.12 (1.73) | 0.75 | 0.89 |  | 39.78 (1.65) | 39.70 (2.45) | 0.92 | 0.94 |
| CBC-MCV | 83.52 (6.13) | 86.85 (4.87) | 0.14 | 0.16 |  | 83.73 (4.96) | 85.21 (4.67) | 0.45 | 0.40 |  | 84.56 (4.91) | 86.26 (5.36) | 0.42 | 0.53 |
| CBC-MCH | 28.87 (2.55) | 29.48 (1.61) | 0.48 | 0.81 |  | 29.22 (2.31) | 29.45 (1.75) | 0.78 | 0.89 |  | 33.69 (15.67) | 30.05 (1.58) | 0.32 | 0.43 |
| CBC-MCHC | 34.54 (0.98) | 33.94 (0.81) | 0.11 | 0.09 |  | 34.88 (1.31) | 34.58 (1.06) | 0.55 | 0.34 |  | 34.49 (1.15) | 34.85 (0.56) | 0.33 | 0.60 |
| CBC-RBC Dist Width | 13.15 (1.09) | 12.45 (0.86) | 0.09 | 0.13 |  | 12.81 (0.73) | 12.85 (1.22) | 0.93 | 0.87 |  | 13.18 (0.84) | 12.80 (0.43) | 0.17 | 0.27 |
| CBC Platelet Count | 274.08 (45.92) | 266.69 (61.32) | 0.74 | 0.53 |  | 284.00 (53.64) | 288.62 (91.64) | 0.88 | 0.74 |  | 300.17 (48.97) | 289.15 (83.78) | 0.69 | 0.45 |
| CBC-Mean Platelet Volume | 7.93 (0.69) | 7.92 (0.75) | 1.00 | 0.98 |  | 7.95 (0.59) | 8.15 (0.73) | 0.45 | 0.34 |  | 8.53 (0.95) | 8.57 (0.87) | 0.90 | 0.85 |

**Table S1c**. **Summary descriptive statistics of clinical assays.** Mean and standard deviation () of the conventional differential blood count profiling in blood at 6, 12 and 24 months following diagnosis with IM. Basophil count was not considered due to a large proportion of missing values.

|  | **6 mo** |  |  |  |  | **12 mo** |  |  |  |  | **24 mo** |  |  |  |
| --- | --- | --- | --- | --- | --- | --- | --- | --- | --- | --- | --- | --- | --- | --- |
| Marker | RC | PI-CFS | p t test | p ranksum |  | RC | PI-CFS | p t test | p ranksum |  | RC | PI-CFS | p t test | p ranksum |
| Monocytes | 6.71 (1.84) | 6.82 (1.86) | 0.88 | 0.85 |  | 5.64 (1.19) | 5.90 (1.02) | 0.57 | 0.76 |  | 5.78 (1.48) | 5.55 (0.83) | 0.63 | 1.00 |
| Lymphocytes | 33.22 (7.73) | 31.52 (7.07) | 0.57 | 0.41 |  | 32.88 (6.66) | 34.43 (9.04) | 0.63 | 0.46 |  | 34.59 (7.67) | 30.12 (5.96) | 0.12 | 0.13 |
| Neutrophils | 54.29 (11.57) | 59.23 (8.51) | 0.23 | 0.25 |  | 57.43 (7.80) | 56.38 (8.72) | 0.75 | 0.53 |  | 54.50 (8.19) | 61.28 (5.68) | *0.02* | *0.01* |
| Eosinophils | 2.31 (1.14) | 2.18 (1.03) | 0.52 | 0.48 |  | 3.08 (1.95) | 2.65 (0.93) | 0.48 | 0.96 |  | 4.17 (3.08) | 2.40 (1.09) | 0.06 | 0.09 |
| Monocyte Absolute | 0.39 (0.08) | 0.45 (0.18) | 0.25 | 0.50 |  | 0.41 (0.10) | 0.38 (0.15) | 0.58 | 0.48 |  | 0.39 (0.09) | 0.39 (0.14) | 0.98 | 0.74 |
| Lymphocytes Absolute | 1.96 (0.61) | 1.98 (0.49) | 0.96 | 0.81 |  | 2.37 (0.39) | 2.15 (0.72) | 0.38 | 0.18 |  | 2.33 (0.42) | 2.06 (0.56) | 0.19 | 0.13 |
| Neutrophils Absolute | 3.47 (1.16) | 4.22 (2.42) | 0.34 | 0.89 |  | 4.54 (2.41) | 3.82 (1.80) | 0.40 | 0.43 |  | 3.86 (1.44) | 4.34 (1.50) | 0.42 | 0.51 |
| Eosinophils Absolute | 0.13 (0.06) | 0.12 (0.05) | 0.44 | 0.60 |  | 0.21 (0.12) | 0.17 (0.09) | 0.31 | 0.28 |  | 0.29 (0.25) | 0.17 (0.10) | 0.21 | 0.20 |
| ESR-Sedimentation Rate | 7.42 (9.37) | 6.77 (3.37) | 0.82 | 0.22 |  | 7.25 (8.21) | 9.00 (8.88) | 0.61 | 0.66 |  | 7.92 (4.19) | 8.58 (6.23) | 1.00 | 0.58 |

**Table S1d**. **Summary descriptive statistics of clinical assays**. Mean and standard deviation () of the conventional endocrine profiling in blood with urine specific gravity and pH at 6, 12 and 24 months following diagnosis with IM.

|  | **6 mo** |  |  |  |  | **12 mo** |  |  |  |  | **24 mo** |  |  |  |
| --- | --- | --- | --- | --- | --- | --- | --- | --- | --- | --- | --- | --- | --- | --- |
| Marker | RC | PI-CFS | p t test | p ranksum |  | RC | PI-CFS | p t test | p ranksum |  | RC | PI-CFS | p t test | p ranksum |
| Triiodothyronine (T3) | 129.16 (36.49) | 151.69 (35.32) | 0.13 | 0.13 |  | 127.42 (24.34) | 143.00 (30.85) | 0.18 | 0.24 |  | 136.36 (41.51) | 140.19 (29.40) | 0.79 | 0.83 |
| Thyroxine (T4) | 7.10 (1.39) | 8.42 (2.00) | 0.07 | 0.10 |  | 7.10 (1.58) | 8.22 (1.47) | 0.08 | 0.08 |  | 8.03 (2.13) | 8.71 (1.81) | 0.39 | 0.25 |
| TSH | 1.90 (0.91) | 1.93 (1.21) | 0.96 | 0.98 |  | 2.35 (1.12) | 2.00 (1.49) | 0.51 | 0.30 |  | 1.88 (0.69) | 2.42 (1.64) | 0.30 | 0.61 |
| ACTH | 18.91 (11.11) | 10.78 (3.58) | *0.01* | *0.02* |  | 32.92 (17.60) | 37.83 (56.12) | 0.21 | 0.10 |  | 34.25 (17.76) | 23.46 (17.71) | 0.14 | 0.18 |
| estradiol | 47.48 (30.45) | 46.92 (66.76) | 0.84 | 0.25 |  | 83.33 (55.22) | 37.67 (31.34) | *0.01* | *0.02* |  | 83.64 (48.84) | 62.92 (56.60) | 0.35 | 0.30 |
| Salivary cortisol (am) | 5.73 (2.96) | 3.68 (1.93) | 0.13 | 0.20 |  | 4.62 (3.26) | 2.19 (1.99) | 0.05 | 0.11 |  | 3.45 (2.35) | 3.37 (1.32) | 0.66 | 0.70 |
| Salivary cortisol (pm) | 0.45 (0.52) | 0.71 (1.21) | 0.81 | 0.92 |  | 0.49 (0.35) | 0.94 (1.21) | 0.48 | 0.66 |  | 0.38 (0.25) | 0.93 (1.10) | 0.16 | 0.36 |
|  |  |  |  |  |  |  |  |  |  |  |  |  |  |  |
| Urine Spec gravity | 1.02 (0.01) | 1.02 (0.01) | 0.53 | 0.39 |  | 1.02 (0.01) | 1.02 (0.01) | 0.36 | 0.35 |  | 1.02 (0.01) | 1.02 (0.01) | 0.18 | 0.30 |
| Urine ph | 6.29 (0.86) | 6.85 (0.66) | 0.08 | 0.12 |  | 5.77 (0.56) | 5.92 (0.73) | 0.81 | 0.84 |  | 6.25 (0.75) | 5.91 (0.66) | 0.09 | 0.06 |
