# Supplementary material for: Tracking post-infectious fatigue in clinic using routine Lab tests
Source: BMC Pediatr. 2016 Apr 26;16:54. doi: 10.1186/s12887-016-0596-8 (PMC4847210; doi:10.1186/s12887-016-0596-8)
Supplement: Additional file 2: Table S2a. — Summary of 2-way ANOVA. Significance of time, group and time x group effects of conventional metabolic profiling in blood across 6, 12 and 24 months following diagnosis with IM. False discovery rates (FDR) were based on Storey [36] using the bootstrap (boot) and the polynomial (poly) fit methods to estimate lambda, as well as on by Benjamini and Hochberg [37] (BH). Table S2b. Summary of 2-way ANOVA. Significance of time, group and time x group effects for complete blood count (CBC) profiling in blood across 6, 12 and 24 months following diagnosis with IM. False discovery rates (FDR) were based on Storey [36] using the bootstrap (boot) and the polynomial (poly) fit methods to estimate lambda, as well as on by Benjamini and Hochberg [37] (BH). Table S2c. Summary of 2-way ANOVA. Significance of time, group and time x group effects for conventional differential blood count profiling in blood across 6, 12 and 24 months following diagnosis with IM. False discovery rates (FDR) were based on Storey [36] using the bootstrap (boot) and the polynomial (poly) fit methods to estimate lambda, as well as on by Benjamini and Hochberg [37] (BH). Basophil count was not considered due to a large proportion of missing values. Table S2d. Summary of 2-way ANOVA. Significance of time, group and time x group effects for standard endocrine profiling in blood with urine specific gravity and pH across 6, 12 and 24 months following diagnosis with IM False discovery rates (FDR) were based on Storey [36] using the bootstrap (boot) and the polynomial (poly) fit methods to estimate lambda, as well as on by Benjamini and Hochberg [37] (BH). (DOC 150 kb) [file 12887_2016_596_MOESM2_ESM.doc]

**Table S2a.** **Summary of 2-way ANOVA**. Significance of time, group and time x group effects of conventional metabolic profiling in blood across 6, 12 and 24 months following diagnosis with IM. False discovery rates (FDR) were based on Storey (2002) using the bootstrap (boot) and the polynomial (poly) fit methods to estimate lambda, as well as on by Benjamini and Hochberg (1995) (BH)

|  | **Time** |  |  |  |  | **Group** |  |  |  |  | **Time x Group** |  |  |  |
| --- | --- | --- | --- | --- | --- | --- | --- | --- | --- | --- | --- | --- | --- | --- |
| Marker | p | FDR boot | FDR poly | FDR_BH |  | p | FDR boot | FDR poly | FDR_BH |  | p | FDR boot | FDR poly | FDR_BH |
| Sodium | 0.86 | 0.06 | 0.15 | 0.91 |  | 0.51 | 0.00 | 0.04 | 0.75 |  | 0.55 | 1.00 | 1.00 | 1.00 |
| Potassium | 0.02 | 0.01 | 0.02 | 0.13 |  | 0.47 | 0.00 | 0.04 | 0.75 |  | 0.73 | 1.00 | 1.00 | 1.00 |
| Chloride | 0.18 | 0.04 | 0.11 | 0.65 |  | 0.25 | 0.00 | 0.03 | 0.56 |  | 0.84 | 1.00 | 1.00 | 1.00 |
| Carbon Dioxide | 0.30 | 0.05 | 0.12 | 0.68 |  | 0.61 | 0.00 | 0.04 | 0.80 |  | 0.86 | 1.00 | 1.00 | 1.00 |
| Glucose | 0.52 | 0.06 | 0.14 | 0.87 |  | 0.51 | 0.00 | 0.04 | 0.75 |  | 0.14 | 1.00 | 1.00 | 1.00 |
| BUN | 0.08 | 0.03 | 0.08 | 0.47 |  | 0.05 | 0.00 | 0.03 | 0.29 |  | 0.85 | 1.00 | 1.00 | 1.00 |
| Creatinine | 0.25 | 0.05 | 0.12 | 0.68 |  | 0.39 | 0.00 | 0.04 | 0.74 |  | 0.84 | 1.00 | 1.00 | 1.00 |
| Calcium | 0.82 | 0.06 | 0.16 | 0.91 |  | 0.14 | 0.00 | 0.02 | 0.37 |  | 0.58 | 1.00 | 1.00 | 1.00 |
| Total Protein, Serum | 0.94 | 0.06 | 0.15 | 0.94 |  | 0.53 | 0.00 | 0.04 | 0.75 |  | 0.20 | 1.00 | 1.00 | 1.00 |
| Albumin | 0.72 | 0.07 | 0.17 | 0.91 |  | 0.58 | 0.00 | 0.04 | 0.78 |  | 0.46 | 1.00 | 1.00 | 1.00 |
| Bilirubin, Total | 0.38 | 0.05 | 0.11 | 0.70 |  | 0.11 | 0.00 | 0.02 | 0.33 |  | 1.00 | 1.00 | 1.00 | 1.00 |
| Alkaline Phosphatase | 0.14 | 0.04 | 0.11 | 0.64 |  | 0.71 | 0.00 | 0.04 | 0.82 |  | 0.76 | 1.00 | 1.00 | 1.00 |
| ALT (SGPT) | 0.72 | 0.07 | 0.16 | 0.91 |  | 0.51 | 0.00 | 0.04 | 0.75 |  | 0.92 | 1.00 | 1.00 | 1.00 |
| AST (SGOT) | 0.70 | 0.07 | 0.18 | 0.91 |  | 0.19 | 0.00 | 0.02 | 0.47 |  | 0.99 | 1.00 | 1.00 | 1.00 |

**Table S2b**. **Summary of 2-way ANOVA**. Significance of time, group and time x group effects for complete blood count (CBC) profiling in blood across 6, 12 and 24 months following diagnosis with IM. False discovery rates (FDR) were based on Storey (2002) using the bootstrap (boot) and the polynomial (poly) fit methods to estimate lambda, as well as on by Benjamini and Hochberg (1995) (BH)

|  | **Time** |  |  |  |  | **Group** |  |  |  |  | **Time x Group** |  |  |  |
| --- | --- | --- | --- | --- | --- | --- | --- | --- | --- | --- | --- | --- | --- | --- |
| Marker | p | FDR boot | FDR poly | FDR_BH |  | p | FDR boot | FDR poly | FDR_BH |  | p | FDR boot | FDR poly | FDR_BH |
| CBC-White Blood cells | 0.32 | 0.04 | 0.11 | 0.68 |  | 0.37 | 0.00 | 0.04 | 0.74 |  | 0.42 | 1.00 | 1.00 | 1.00 |
| CBC-Red Blood Cells | 0.34 | 0.04 | 0.11 | 0.68 |  | 0.13 | 0.00 | 0.02 | 0.36 |  | 1.00 | 1.00 | 1.00 | 1.00 |
| CBC-Hemoglobin | 0.37 | 0.05 | 0.12 | 0.70 |  | 0.85 | 0.00 | 0.04 | 0.87 |  | 0.79 | 1.00 | 1.00 | 1.00 |
| CBC-Hematocrit | 0.71 | 0.07 | 0.18 | 0.91 |  | 0.84 | 0.00 | 0.04 | 0.87 |  | 0.77 | 1.00 | 1.00 | 1.00 |
| CBC-MCV | 0.80 | 0.06 | 0.16 | 0.91 |  | 0.07 | 0.00 | 0.02 | 0.29 |  | 0.79 | 1.00 | 1.00 | 1.00 |
| CBC-MCH | 0.27 | 0.05 | 0.11 | 0.68 |  | 0.54 | 0.00 | 0.04 | 0.75 |  | 0.45 | 1.00 | 1.00 | 1.00 |
| CBC-MCHC | 0.18 | 0.04 | 0.11 | 0.65 |  | 0.45 | 0.00 | 0.04 | 0.75 |  | 0.23 | 1.00 | 1.00 | 1.00 |
| CBC-RBC Dist Width | 0.73 | 0.06 | 0.16 | 0.91 |  | 0.10 | 0.00 | 0.02 | 0.33 |  | 0.36 | 1.00 | 1.00 | 1.00 |
| CBC Platelet Count | 0.44 | 0.05 | 0.12 | 0.76 |  | 0.77 | 0.00 | 0.04 | 0.85 |  | 0.91 | 1.00 | 1.00 | 1.00 |
| CBC-Mean Platelet Volume | 0.01 | 0.01 | 0.03 | 0.13 |  | 0.65 | 0.00 | 0.04 | 0.81 |  | 0.89 | 1.00 | 1.00 | 1.00 |

**Table S2c**. **Summary of 2-way ANOVA**. Significance of time, group and time x group effects for conventional differential blood count profiling in blood across 6, 12 and 24 months following diagnosis with IM. False discovery rates (FDR) were based on Storey (2002) using the bootstrap (boot) and the polynomial (poly) fit methods to estimate lambda, as well as on by Benjamini and Hochberg (1995) (BH). Basophil count was not considered due to a large proportion of missing values.

|  | **Time** |  |  |  |  | **Group** |  |  |  |  | **Time x Group** |  |  |  |
| --- | --- | --- | --- | --- | --- | --- | --- | --- | --- | --- | --- | --- | --- | --- |
| Marker | p | FDR boot | FDR poly | FDR_BH |  | p | FDR boot | FDR poly | FDR_BH |  | p | FDR boot | FDR poly | FDR_BH |
| Monocytes | 0.01 | 0.01 | 0.02 | 0.13 |  | 0.89 | 0.00 | 0.04 | 0.89 |  | 0.82 | 1.00 | 1.00 | 1.00 |
| Lymphocytes | 0.78 | 0.06 | 0.16 | 0.91 |  | 0.37 | 0.00 | 0.04 | 0.74 |  | 0.36 | 1.00 | 1.00 | 1.00 |
| Neutrophils | 0.88 | 0.06 | 0.15 | 0.91 |  | 0.08 | 0.00 | 0.01 | 0.29 |  | 0.25 | 1.00 | 1.00 | 1.00 |
| Eosinophils | 0.11 | 0.04 | 0.09 | 0.56 |  | 0.05 | 0.00 | 0.02 | 0.29 |  | 0.21 | 1.00 | 1.00 | 1.00 |
| Monocyte Absolute | 0.77 | 0.07 | 0.17 | 0.91 |  | 0.66 | 0.00 | 0.04 | 0.81 |  | 0.42 | 1.00 | 1.00 | 1.00 |
| Lymphocytes Absolute | 0.15 | 0.04 | 0.10 | 0.64 |  | 0.22 | 0.00 | 0.03 | 0.50 |  | 0.63 | 1.00 | 1.00 | 1.00 |
| Neutrophils Absolute | 0.80 | 0.06 | 0.16 | 0.91 |  | 0.69 | 0.00 | 0.04 | 0.82 |  | 0.34 | 1.00 | 1.00 | 1.00 |
| Eosinophils Absolute | 0.03 | 0.01 | 0.03 | 0.21 |  | 0.05 | 0.00 | 0.02 | 0.29 |  | 0.27 | 1.00 | 1.00 | 1.00 |
| ESR-Sedimentation Rate | 0.82 | 0.06 | 0.15 | 0.91 |  | 0.72 | 0.00 | 0.04 | 0.82 |  | 0.84 | 1.00 | 1.00 | 1.00 |

**Table S2d**. **Summary of 2-way ANOVA**. Significance of time, group and time x group effects for standard endocrine profiling in blood with urine specific gravity and pH across 6, 12 and 24 months following diagnosis with IM False discovery rates (FDR) were based on Storey (2002) using the bootstrap (boot) and the polynomial (poly) fit methods to estimate lambda, as well as on by Benjamini and Hochberg (1995) (BH).

|  | **Time** |  |  |  |  | **Group** |  |  |  |  | **Time x Group** |  |  |  |
| --- | --- | --- | --- | --- | --- | --- | --- | --- | --- | --- | --- | --- | --- | --- |
| Marker | p | FDR boot | FDR poly | FDR_BH |  | p | FDR boot | FDR poly | FDR_BH |  | p | FDR boot | FDR poly | FDR_BH |
| Triiodothyronine (T3) | 0.86 | 0.06 | 0.15 | 0.91 |  | 0.07 | 0.00 | 0.02 | 0.29 |  | 0.61 | 1.00 | 1.00 | 1.00 |
| Thyroxine (T4) | 0.31 | 0.05 | 0.12 | 0.68 |  | 0.01 | 0.00 | 0.01 | 0.26 |  | 0.81 | 1.00 | 1.00 | 1.00 |
| TSH | 0.72 | 0.07 | 0.18 | 0.91 |  | 0.80 | 0.00 | 0.04 | 0.87 |  | 0.44 | 1.00 | 1.00 | 1.00 |
| ACTH | 0.01 | 0.01 | 0.02 | 0.11 |  | 0.01 | 0.00 | 0.03 | 0.26 |  | 0.95 | 1.00 | 1.00 | 1.00 |
| estradiol | 0.21 | 0.04 | 0.11 | 0.65 |  | 0.06 | 0.00 | 0.02 | 0.29 |  | 0.30 | 1.00 | 1.00 | 1.00 |
| Salivary cortisol (am) | 0.22 | 0.04 | 0.11 | 0.65 |  | 0.03 | 0.00 | 0.02 | 0.29 |  | 0.28 | 1.00 | 1.00 | 1.00 |
| Salivary cortisol (pm) | 0.89 | 0.06 | 0.15 | 0.91 |  | 0.06 | 0.00 | 0.02 | 0.29 |  | 0.85 | 1.00 | 1.00 | 1.00 |
|  |  |  |  |  |  |  |  |  |  |  |  |  |  |  |
| Urine Spec gravity | 0.32 | 0.05 | 0.12 | 0.68 |  | 0.10 | 0.00 | 0.02 | 0.33 |  | 0.95 | 1.00 | 1.00 | 1.00 |
| Urine ph | 0.00 | 0.01 | 0.02 | 0.11 |  | 0.49 | 0.00 | 0.04 | 0.75 |  | 0.10 | 1.00 | 1.00 | 1.00 |
